# Effect of inactivated *Streptococcus pneumoniae* as non-pathogenic particles on the severity of pneumonia caused by respiratory syncytial virus infection in mice

**DOI:** 10.1016/j.toxrep.2019.05.004

**Published:** 2019-05-31

**Authors:** Aki Miyauchi, Wataru Watanabe, Toshi Akashi, Seiko Hashiguchi, Hiroki Yoshida, Chihiro Sugita, Masahiko Kurokawa

**Affiliations:** a Departments of Biochemistry and Microbiology, Graduate School of Clinical Pharmacy, Kyushu University of Health and Welfare, 1714-1 Yoshino, Nobeoka, Miyazaki 882-8508, Japan; b Department of Microbiology and Infectious Diseases, School of Pharmaceutical Sciences, Kyushu University of Health and Welfare, 1714-1 Yoshino, Nobeoka, Miyazaki 882-8508, Japan

**Keywords:** RSV, respiratory syncytial virus, *S. pneumoniae*, *Streptococcus pneumoniae*, TiO_2_, titanium dioxide, IFN, interferon, BALF, bronchoalveolar lavage fluids, ISP, inactivated *S. pneumoniae*, PFU, plaque-forming units, PBS, phosphate-buffered saline, CFU, colony-forming units, ELISA, enzyme-linked immunosorbent assay, RSV, *Streptococcus pneumonia*, Pneumonia, Infiltrated cells, Non-pathogenic pneumococcal particles

## Abstract

The severity of pneumonia in respiratory syncytial virus (RSV) infection is strongly related to host immune response and external factors such as bacteria and environmental chemicals. We investigated the effect of inactivated *Streptococcus pneumoniae* (ISP) as non-pathogenic particles on the severity of pneumonia in RSV-infected mice. Mice were intranasally exposed to ISP before RSV infection. On day 5 post-infection, we examined tissues, virus titer, and infiltrated cells in the lungs. The ISP did not cause significant histopathological effects in the lungs of RSV infected mice, but reduced virus titer. It also reduced the ratio of lymphocyte infiltration into the lungs and consequently the ratio of macrophage increased. In addition, we found that ISP increased RANTES level in bronchoalveolar lavage fluid from RSV-infected mice on day 1 post-infection, but reduced type I interferon levels. Thus, ISP did not exacerbate pneumonia in RSV infection, rather, it might mildly reduce the severity. We characterize and discuss the inherent activity of ISP as non-pathogenic particles inducing the role of RANTES on the pneumonia in RSV infection.

## Highlights

• We made inactivated *Streptococcus pneumoniae* (ISP) as non-pathogenic particles.
• We evaluated effects of ISP on development of pneumonia by RSV infection in mice.
• ISP didn’t show histopathological effects on lungs of RSV-infected mice.
• ISP reduced virus titer and infiltration of lymphocyte in the lungs.
• The inherent activity of ISP as particles in RSV infection is discussed.

## Graphical abstract

**Figure:**
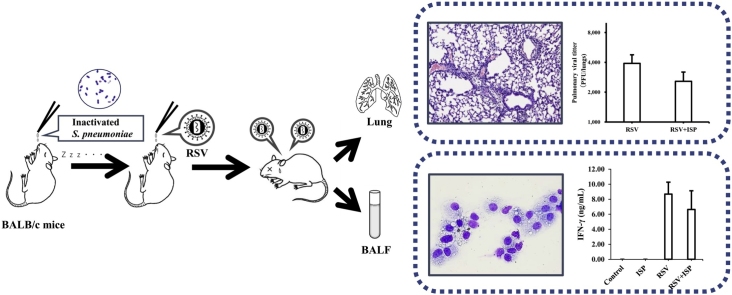

## 1 Introduction

Respiratory syncytial virus (RSV, *Paramyxoviridae* family) causes severe respiratory infections in infants, young children, and the elderly worldwide [[1], [2], [3]], and mass infection at schools and hospitals due to contact infection in winter [4]. Symptoms of RSV infection have been demonstrated to develop mainly as a result of host immune response, and RSV interaction with the host immune system is crucial in determining the outcome in diseases such as pneumonia [5]. Also, external factors such as bacteria and environmental chemical substances are thought to be one of the aggravating factors of pneumonia in RSV infection [[6], [7], [8]]. *Streptococcus pneumoniae* (*S. pneumoniae*) is known as the major cause of death among all respiratory pathogens and causes not only respiratory infections such as pneumonia and sinusitis but also meningitis or septicemia. It has been reported that many healthy children and adults possess *S. pneumoniae* in the upper respiratory tract [9]. In the nasopharynx of 45% of children under 3 years of age and up to 20% of adults, *S. pneumoniae* is colonized [9]. Clinically, concurrent infection with RSV and *S. pneumoniae* was suggested to cause severe pneumonia [10,11]. In mice, concurrent infection by RSV and *S. pneumoniae* has been also reported to cause severer pneumococcaemia than their single infection [12]. *In vitro*, *S. pneumoniae* has been shown to adhere to RSV-infected human epithelial cells and bind directly to RSV [12,13]. Thus, the severity of RSV infection is suggested to closely relate to the existence of *S. pneumoniae in vivo*. However, it is unclear how the concurrent infection of RSV and *S. pneumoniae* exacerbate pneumonia.

Non-microbial substances, such as nanomaterials and irritating gas, have been shown to damage lungs and trachea [7,14]. These substances are also possible factors to exacerbate pneumonia in RSV infection. We previously evaluated the effects of TiO_2_ nanoparticles, an environmental chemical, on RSV infection in mice [7]. In that report, the exposure of mice to TiO_2_ nanoparticles enhanced the levels of interferon (IFN)-γ and chemokine RANTES, representative markers of pneumonia, in the bronchoalveolar lavage fluids (BALF) of RSV-infected mice and histopathologically exacerbated pneumonia in RSV-infected mice [7]. The immune system of RSV-infected mice has been shown to be significantly affected by TiO_2_ nanoparticles as non-pathogenic particles, resulting in the exacerbation of pneumonia caused by RSV infection. This suggested that non-infectious bacteria particles are capable of affecting host immune response in RSV infection. Thus, it is possible that *S. pneumoniae,* as a non-pathogenic particle itself, affects the severity of pneumonia in RSV infection.

In the present study, we inactivated *S. pneumoniae* by formalin and investigated its effect as non-pathogenic particles on the severity of pneumonia in RSV infection in mice to assess a mode of exacerbation of pneumonia in RSV infection by the concurrent infection with *S. pneumoniae.* Mice were intranasally exposed to the inactivated *S. pneumoniae* (ISP) every other day for five days, and then RSV was infected intranasally. On day 1 or 5 post RSV infection, we histopathologically examined lung tissues and immune cells in BALF prepared from RSV-infected and uninfected mice, and also evaluated the levels of IFNs and RANTES in the BALF. We characterized the inherent activity of the ISP as non-pathogenic particles on pneumonia in RSV infection.

## 2 Materials and methods

### 2.1 Animals

We used female (5 weeks old) BALB/c mice purchased from Kyudo Animal Laboratory (Kumamoto, Japan). The mice were housed at five to six per cage under a 12 h light/dark cycle at 25 ± 2 °C. They were fed a standard solid diet (CRF-1, Oriental Yeast Co., Chiba, Japan), given water *ad libitum,* and acclimated for 7 d before experiments. Experimental protocols were approved by the Animal Experiment Committee of Kyushu University of Health and Welfare, Japan (approval numbers: 27-1-31 and 28-1-06) and the animal experimentation guidelines were followed in animal studies.

### 2.2 Virus and cell

A2 strain of RSV was obtained from American Type Culture Collection (Rockville, MD, USA). Human epidermoid carcinoma HEp-2 cells (American Type Culture Collection CCL-23) were purchased from Dainippon Pharmaceutical (Osaka, Japan). HEp-2 cells were maintained in Eagle’s minimum essential medium supplemented with heat-inactivated 10% fetal calf serum. RSV was grown in HEp-2 cell cultures and viral titers were measured by a plaque method [15]. The virus yields were expressed as plaque-forming units per milliliter (PFU/mL) [15]. *In vivo* experiments, HEp-2 cells were used for titration of the virus yield in the lungs of mice.

### 2.3 *S. pneumoniae* and its inactivation

*S. pneumoniae* (ATCC49619, serotype 19F) was purchased from American Type Culture Collection (Rockville, MD, USA) and grown on 5% sheep blood agar medium at 37 °C. The *S. pneumoniae* grown in the agar medium were collected and suspended in phosphate-buffered saline (PBS). The concentration of *S. pneumoniae* in the suspension was measured by counting colony-forming units (CFU). The *S. pneumoniae* suspension at 1.0 × 10^8^ CFU/mL was centrifuged at 2150 × *g*, then the precipitated *S. pneumoniae* was suspended and inactivated in 2% formalin solution for 60 h at room temperature. The ISP was centrifuged at 2150 × *g*, and then the precipitated ISP was twice rinsed with PBS. The rinsed *S. pneumoniae* was suspended in PBS and stored at −30 °C. Before animal experiments, the stored ISP was rinsed three times with PBS and suspended in PBS at 1.0 × 10^8^ CFU/mL. The suspension of 0.1 mL was used for the intranasal exposure of mice. The remaining formalin in the ISP suspension was determined by Schiff’s reagent according to the manufacturer’s instructions (Nacalai Tesque, Kyoto, Japan).

### 2.4 Animal tests

Mice were intranasally exposed to 0.1 mL of a suspension of ISP at 1.0 × 10^8^ CFU/mL once daily on days 1, 3, and 5 before RSV infection under anesthesia with ketamine and xylazine at 40 and 6 μg/kg of body weight, respectively. In the control group, mice were intranasally exposed to PBS (0.1 mL) under anesthesia. RSV (1.0 × 10^6^ PFU/100 μL) was intranasally infected to the ISP-exposed mice under anesthesia [15]. In the uninfected group, mice were intranasally given PBS. On day 1 or 5 post-infection, lungs and BALF from the mice were prepared under anesthesia.

### 2.5 Histopathological methods

For histopathological examination of lungs, lungs were removed from mice on day 5 post-infection under anesthesia. Briefly, 10% buffered formalin solution was injected into lungs *via* trachea and then the removed lungs were immersed in the solution and fixed. The fixed tissue was then embedded in paraffin, sectioned at a thickness of 4 μm, and stained with hematoxylin and eosin. The samples were observed under a microscope (×100) and scored. To score the inflammation of the lungs, 4 lobes of the lung sections were quantitatively analyzed. The degree of thickness of the alveolar wall, infiltration of inflammatory cells into alveoli, and lymphocyte infiltration around the pulmonary artery were graded on a scale of 0–4 (0, absent; 1, mild; 2, moderate; 3, severe; 4, very severe) as described previously [16]. The total histopathological lung inflammation score comprises the sum of the scores for four lobes (maximum score is 16). In each group, the lungs of two to five mice (control and ISP-exposed mice without RSV infection: n = 2, RSV-infected mice with and without exposure of ISP: n = 5) were evaluated, and the average value was calculated.

### 2.6 IFNs and RANTES levels in BALF

BALF was prepared from RSV-infected and uninfected mice on day 1 and day 5 post-infection. Briefly, BALF was obtained from the mice under anesthesia by instilling 0.8 mL of cold PBS into the lungs and aspirating it from the trachea using a tracheal cannula [15]. The obtained BALF was centrifuged at 160 × *g* at 4 °C for 10 min. The supernatant was stored at −80 °C until the use for enzyme-linked immunosorbent assay (ELISA). The levels of IFN-α, IFN-β, IFN-γ, and RANTES (CCL5) in BALF were measured using ELISA kits (VeriKine, PBL Assay Science., Piscataway, NJ, USA; Quantikine, R&D Systems, Inc., Minneapolis, MN, USA; Ready-set-go, eBioscience Inc., San Diego, CA, USA; and Quantikine, R&D Systems, Inc., Minneapolis, MN, USA; respectively) according to the manufacturer’s instructions. The lower limits of detection of the kits are 12.5 pg/mL for IFN-α, 1.89 pg/mL for IFN-β, 15 pg/mL for IFN-γ, and 2 pg/mL for RANTES. The intra- and inter-assay coefficients of variation for the ELISA results were less than 10%.

### 2.7 Virus titration

Virus yields in the lungs were determined in RSV-infected mice. The lungs were removed under anesthesia on day 5 after infection. The removed lungs were immediately frozen in liquid N_2_ and stored at −80 °C until use. The frozen lungs were homogenized with cold quartz sand in a homogenizer [15]. The homogenate was centrifuged at 480 × *g* at 4 °C for 15 min. Virus yield [PFU/lung (n = 5)] in the supernatant was determined by the plaque assay on Hep-2 cells [15,17].

### 2.8 Analysis of bronchoalveolar lavage cells

Bronchoalveolar lavage cells were collected from each BALF by centrifugation at 160 × *g* for 10 min. The collected cells were suspended in PBS, counted using a hemocytometer, and then expressed as the total cell number per μL of each BALF. Also, the cells were smeared onto slides, stained with Wright-Giemsa staining solution (Muto Pure Chemicals Co., Ltd., Tokyo, Japan) for 3–4 min, and then washed with water until the edge was faintly pinkish red. Finally, the cell morphology was histopathologically observed under a microscope (×400), and macrophages, neutrophils, and lymphocytes were identified and counted. Their ratios were expressed as percentages of 200 cells per specimen (control: n = 2, ISP-exposed mice without RSV infection: n = 3, others: n = 6).

### 2.9 Statistical analysis

Comparisons of the histopathological scores of the lungs, the levels of IFNs and RANTES in BALF, the pulmonary viral titers, and the percentages of bronchoalveolar lavage cells were carried out using the Student’s *t*-test. A value of *P* <  0.05 or less was considered statistically significant.

## 3 Results

### 3.1 Effect of ISP on severity of pneumonia by RSV infection

*S. pneumoniae* was inactivated by formalin to assess the severity of pneumonia in RSV infection by *S. pneumoniae* as non-pathogenic particles. The ISP was suspended in PBS, and mice were intranasally exposed to the ISP once daily on days 1, 3, and 5 before RSV infection. The inactivation of *S. pneumoniae* was confirmed by a growth assay on blood agar medium, and no morphological changes of *S. pneumoniae* by formalin treatment were observed under a microscope (data not shown). When examined by Schiff’s reagent, no remaining formalin was detected (data not shown). Thus, it was confirmed that *S. pneumoniae* is completely inactivated and the toxicity of the remaining formalin in suspension was excluded. In fact, no abnormal behavior or dystrophy of mice was observed after exposure of the ISP in mice.

**Fig. 1:**
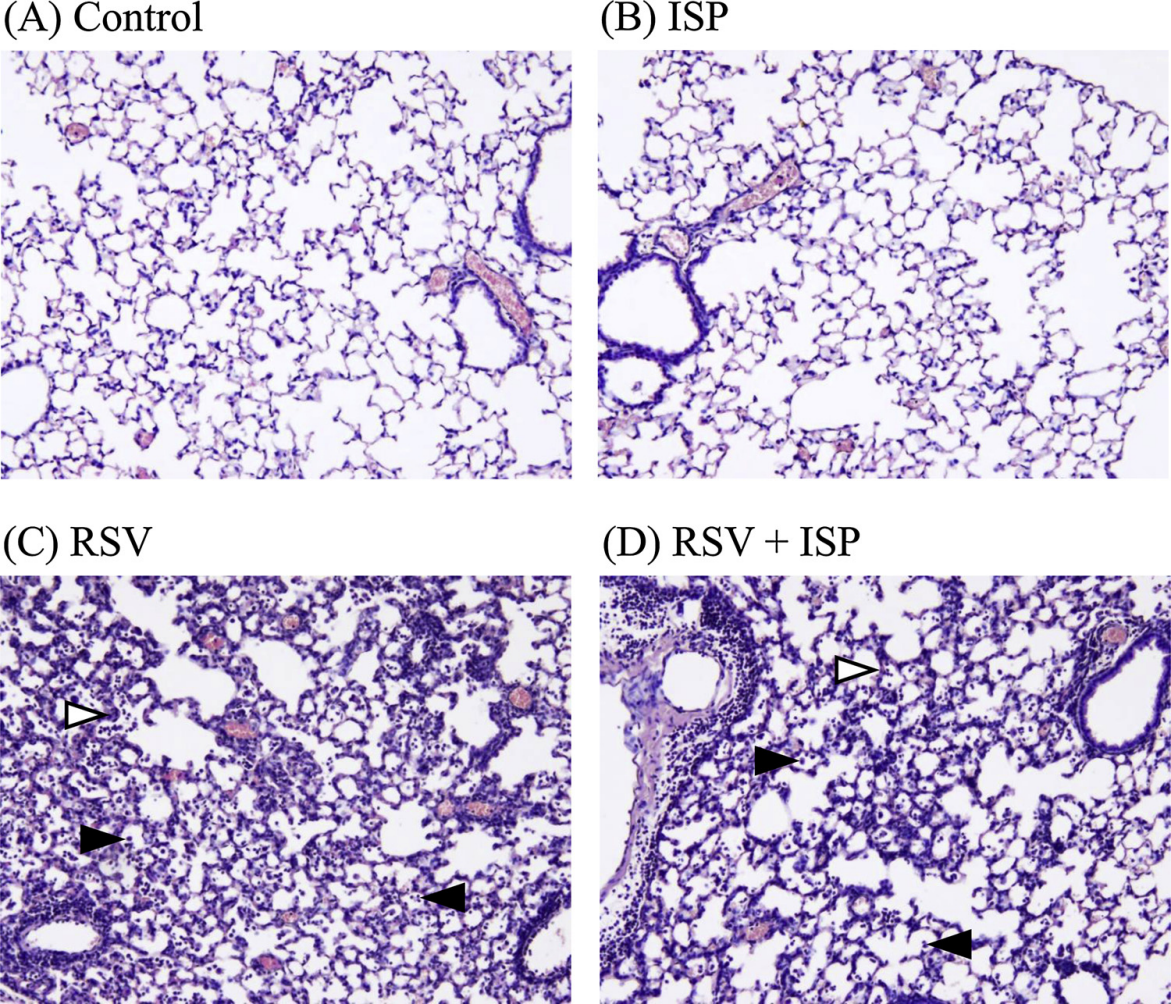
Histopathology of lung tissues of RSV-infected mice exposed to ISP. On day 5 post RSV infection, lungs were prepared from RSV-uninfected (A: control) and -infected (C: RSV) mice, and also from ISP-exposed mice to (D: RSV + ISP) and not (B: ISP) RSV infection. The lung tissues were stained with hematoxylin and eosin, and then observed under a microscope (× 100). Open arrowheads indicate thickened alveolar walls. Closed arrowheads indicate mononuclear cells infiltrated into alveoli.

Mice exposed or unexposed to the ISP were intranasally infected with RSV at 0 or 1.0 × 10^6^ PFU. On day 5 post-infection, the lung tissues of RSV-infected mice were histopathologically analyzed. Fig. 1 shows representative lung tissues of mice exposed or not exposed to ISP with or without RSV infection. In the exposed mice without RSV infection, no obvious changes in the lung tissues due to ISP were observed, as well as in the unexposed mice without RSV infection (Fig. 1A and B). On the other hand, in the unexposed mice with RSV infection, typical features of pneumonia, such as degeneration of the bronchial epithelium, infiltration of lymphocytes, and neutrophils, were observed (Fig. 1C). Similar features of pneumonia were also observed in ISP-exposed mice with RSV infection, but no noticeable differences were observed between RSV-infected mice with and without ISP exposure (Fig. 1C and D).

We quantitatively compared the severity of pneumonia in the four lung lobes of each RSV-infected or -uninfected mouse with or without exposure to ISP. As indexes of pneumonia, the thickness of the alveolar wall, infiltration of inflammatory cells into alveoli, and infiltration of lymphocytes around the pulmonary artery were scored. As shown in Table 1, although the indexes in RSV-infected mice with exposure to ISP were lower than those in RSV-infected mice without the exposure, there were no significant differences. In RSV-uninfected mice with and without the exposure, histopathological changes due to the exposure to ISP were not significantly observed. Thus, no obvious exacerbation of pneumonia in RSV-infected mice was histopathologically observed due to exposure to ISP.

**Table 1:** Lung inflammation score of mice on day 5 after RSV infection.

Evaluation item	Inflammation score^a^			
	Control	ISP	RSV	RSV + ISP
Thickness of alveolar wall	0	0	8.6 ± 1.02	8.0 ± 1.10
Infiltration of inflammatory cells into alveoli	0	2.0	8.2 ± 0.75	7.4 ± 1.36
Lymphocyte infiltration around pulmonary artery	0	0	8.2 ± 0.75	7.2 ± 1.17

Control = RSV-uninfected mice; ISP = ISP-exposed mice without RSV infection; RSV = RSV-infected mice; RSV + ISP = ISP-exposed mice with RSV infection.

a Lung tissues were scored in 5 grades of 0–4 (0, absent; 2, moderate; 3, severe; 4, very severe) for each of 4 lobes, and the extent of inflammation was evaluated by totaling them. Data represents mean ± standard deviation of 2 or 5 mice (Control and ISP, n = 2 and RSV and RSV + ISP, n = 5).

### 3.2 Effect of ISP on IFN-γ production in BALF from RSV-infected mice

**Fig. 2:**
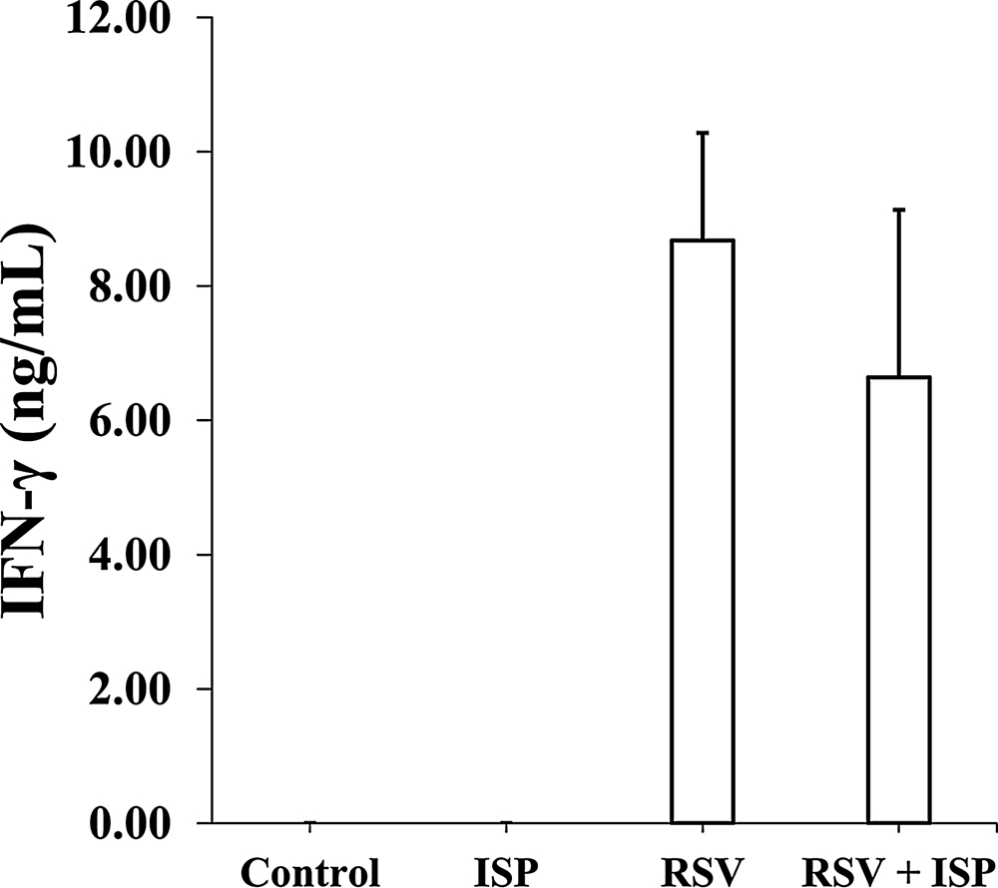
Effect of ISP on IFN-γ production in BALF of RSV-infected and -uninfected mice. Mice were intranasally exposed to ISP and then infected with RSV. On day 5 post-infection, BALF was prepared, and the IFN-γ concentration in the BALF was determined by ELISA as described in Materials and Methods. Control, RSV-uninfected mice; ISP, ISP-exposed mice without RSV infection; RSV, RSV-infected mice; RSV + ISP, ISP-exposed mice with RSV infection. The data represent mean ± standard deviation of values of 2–6 mice (control: n = 2, others: n = 6/group).

IFN-γ, a Th1 cytokine, in BALF of RSV-infected mice is a sensitive marker of the severity of pneumonia by RSV infection [18]. As shown in Fig. 2, we compared IFN- γ levels in the BALF prepared from RSV-infected mice with and without the exposure of ISP on day 5 post RSV infection. In the RSV-uninfected mice, the IFN-γ levels were not detected even by the exposure to ISP. On the other hand, in the RSV-infected mice without the exposure of ISP, IFN-γ level increased remarkably as reported previously [6,7,15]. Although the exposure of ISP slightly reduced the IFN-γ level as compared with that in RSV-infected mice without the exposure, the slight reduction was not statistically significant. Thus, it was suggested that the exposure of ISP did not affect the exacerbation of pneumonia in RSV infection.

### 3.3 Influence of ISP on virus titer in the lungs of RSV-infected mice

**Fig. 3:**
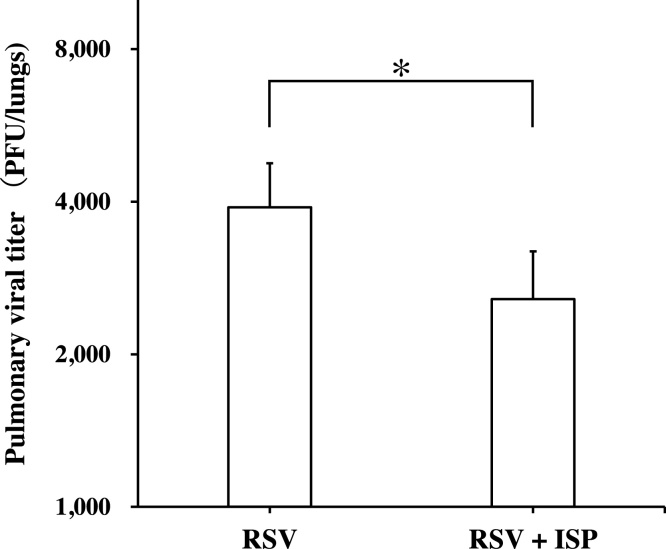
Effect of ISP exposure on RSV yield in the lungs of RSV-infected mice on day 5 post-infection. RSV titers in the lungs were determined by plaque assay. RSV, RSV-infected mice; RSV + ISP, mice with RSV infection exposed to ISP. The data represents mean ± standard deviation of 5 mice/group. *Significantly different from control at *P* <  0.05 (Student’s *t*-test).

The virus titer in the lungs of RSV-infected mice has been shown to correlate with the severity of pneumonia caused by RSV infection [8,15]. We compared the viral titers of lungs prepared from RSV-infected mice with and without the exposure to ISP on day 5 post RSV infection. As shown in Fig. 3, the exposure to ISP significantly reduced the virus titer of lungs of RSV-infected mice. Thus, ISP was suggested to be effective in alleviating pneumonia in RSV-infected mice. However, this result did not sufficiently reflect the histopathological results, as shown in Fig. 1 and Table 1.

### 3.4 Effect of ISP on infiltrated cells in the lungs of RSV-infected mice

**Fig. 4:**
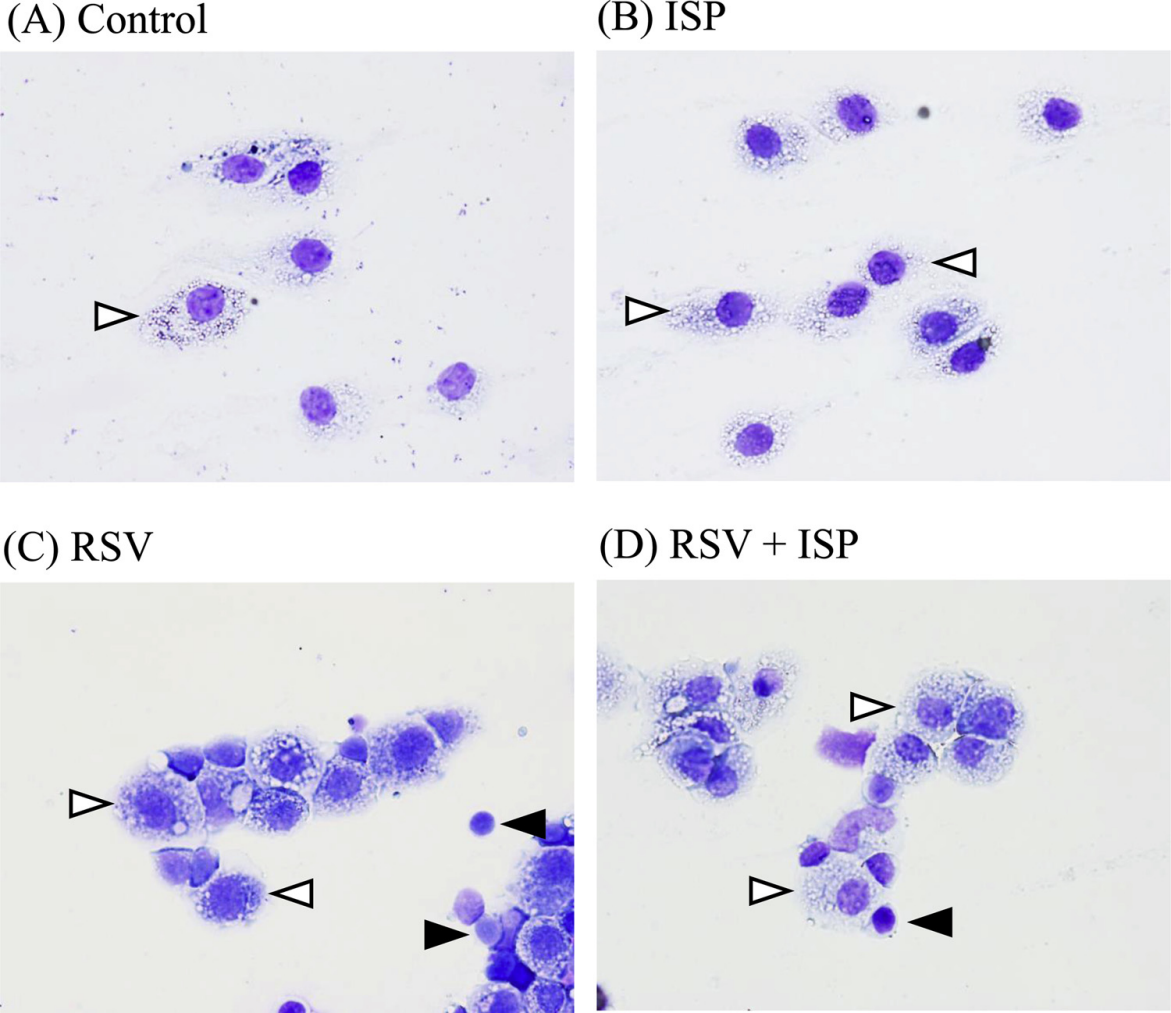
Histopathology of infiltrated cells in BALF of RSV-infected mice with and without the exposure to ISP. On day 5 after RSV infection, BALF was prepared from RSV-uninfected (A: control) and -infected (C: RSV) mice, and also from ISP-exposed mice with (D: RSV + ISP) and without (B: ISP) RSV infection. Bronchoalveolar lavage cells were stained with Wright-Giemsa solution and then observed under a microscope (×400). Open arrowheads indicate mononuclear lineage cells. Closed arrowheads indicate lymphocytes.

RSV interaction with the host immune system is crucial in deteriorating pneumonia [5,6,15]. To estimate the immune status of lungs of RSV-infected mice exposed to ISP, we histopathologically characterized and compared the infiltrated cells in BALF prepared from RSV-infected mice with and without exposure to ISP. Fig. 4 shows the representative infiltrated cells in the BALF of RSV-infected or uninfected mice exposed or not to ISP. In the RSV-uninfected mice exposed (Fig. 4B) or not exposed (Fig. 4A) to ISP, most cells observed were mononuclear lineage cells regardless of the exposure, and the exposure to ISP did not remarkably affect the population of infiltrated cells in RSV-uninfected mice. In the BALF of RSV-infected mice without exposure to ISP (Fig. 4C) the population of lymphocytes increased, and the population seemed to be larger than that in the BALF of RSV-infected mice with exposure to ISP (Fig. 4D). In the RSV-infected mice exposed to ISP (Fig. 4D), the ratio of mononuclear lineage cells in the infiltrated cells seemed to be larger than that in the RSV-infected mice without the exposure.

We histopathologically identified lymphocytes, macrophages, neutrophils, and the other cells as cells infiltrated into BALF and examined their ratios in total cells in BALF, as shown in Table 2. In the RSV-uninfected mice without the exposure to ISP, the total number of infiltrated cells was very low (91.7 ± 8.3 cells/μL), but the exposure to ISP noticeably increased the total number of infiltrated cells (327.8 ± 92.2 cells/μL). In the either case, most infiltrated cells were macrophages (100% and 99.8 ± 0.2%). However, in RSV-infected cells with and without the exposure, the total numbers of infiltrated cells (784.7 ± 223.4 and 972.2 ± 168.0 cells/μL, respectively) were similar, and their numbers were larger than that in the RSV-uninfected mice with the exposure (327.8 ± 92.2 cells/μL). In RSV-infected mice with and without the exposure, the large populations of infiltrated cells were lymphocytes and macrophages, and the ratios of neutrophils and other cells were very low. The actual numbers of macrophages in RSV-infected mice with (541 cells/μL) and without (465 cells/μL) the exposure were similar and slightly higher than that in RSV-uninfected mice with the exposure (327 cells/μL). In RSV-infected mice, the exposure reduced the ratio of lymphocytes in the infiltrated cells but remarkably increased that of macrophages. Thus, it was possible that ISP mainly interfered with the infiltration of lymphocytes into the lungs of RSV-infected mice, resulting in the rise of ratio of macrophage population. As a result, the exposure to ISP was effective in reducing lymphocyte infiltration into the lungs of RSV-infected mice.

**Table 2:** Analysis of bronchoalveolar lavage cells on day 5 after RSV infection.

Treatment	Lymphocytes (%)	Macrophages (%)	Neutrophil (%)	Others (%)	Total cell number/μL
Control	0	100	0	0	91.7 ± 8.3
ISP	0.2 ± 0.2	99.8 ± 0.2	0	0	327.8 ± 92.2
RSV	49.7 ± 6.8	47.8 ± 7.0	2.5 ± 1.8	0	972.2 ± 168.0
RSV + ISP	27.9 ± 0.2*	68.9 ± 6.3*	3.2 ± 1.6	0	784.7 ± 223.4

Control = RSV-uninfected mice; ISP = ISP-exposed mice without RSV infection; RSV = RSV-infected mice; RSV + ISP = ISP-exposed mice with RSV infection.

Data represent mean ± standard deviation of 2–6 mice (Control, n = 2; ISP, n = 3; RSV and RSV + ISP, n = 6).

* Statistically different from RSV-infected group at *P* < 0.01 by Student’s *t*-test.

### 3.5 Effect of ISP on the levels of type I IFNs and RANTES in BALF from RSV-infected mice

**Fig. 5:**
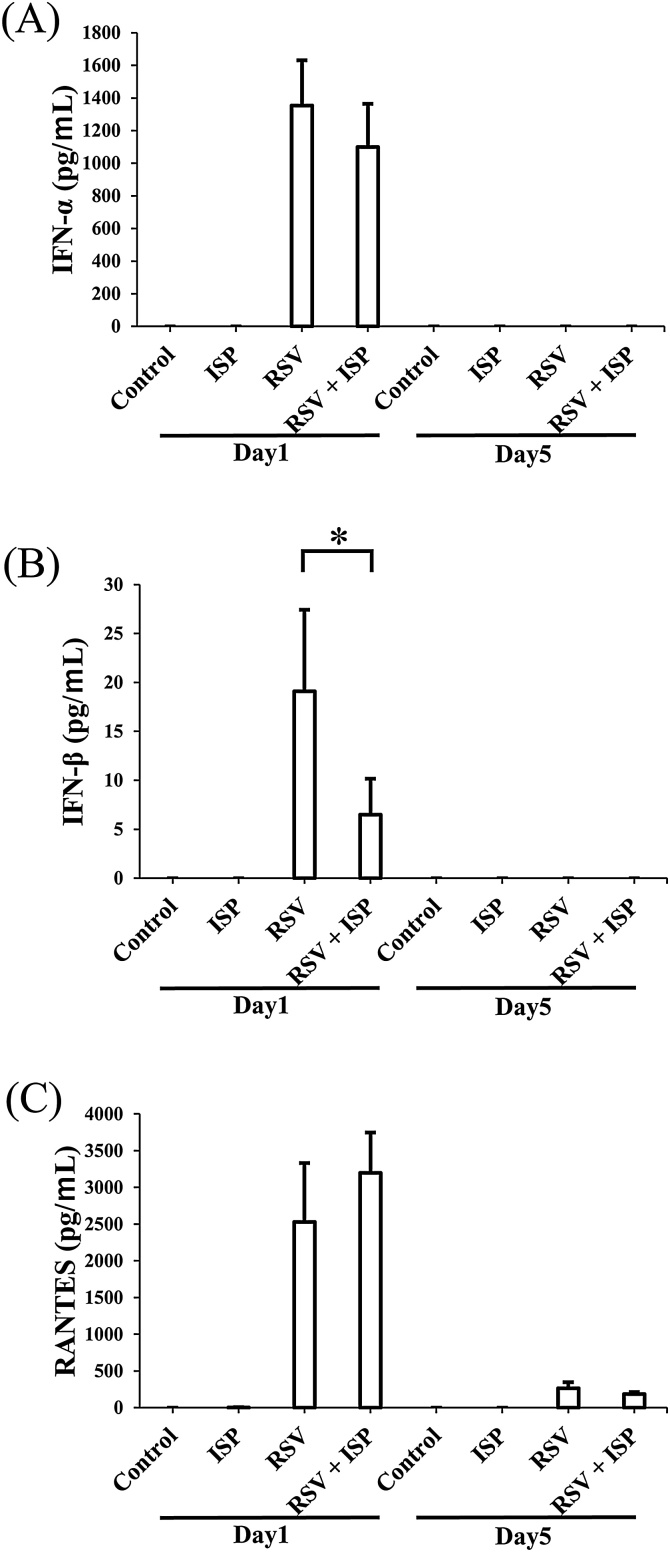
Effect of ISP on type I IFNs and RANTES productions in BALF of RSV-infected and uninfected mice. Mice were intranasally exposed to ISP and then infected with RSV. On day 1 and day 5 post-infection, BALF was prepared, and the IFN-α (A) and β (B) and RANTES (C) concentrations in the BALF were determined by ELISA as described in Materials and Methods. Control, RSV-uninfected mice; ISP, ISP-exposed mice without RSV infection; RSV, RSV-infected mice; RSV + ISP, ISP-exposed mice with RSV infection. The data represent mean ± standard deviation of values of 2 or 6 mice (control: n = 2, others: n = 6/group).

Type I IFNs are important immune mediators with antiviral activity, have been shown to be produced by live *S. pneumoniae*-infected macrophages, and positively regulate RANTES production in macrophages and cocultured alveolar epithelial cells *in vitro* [19]. Also, type I IFNs have been shown to control RANTES production during pneumococcal pneumonia *in vivo* [19]. Further, RANTES has been shown to facilitate macrophage infiltration in the early phase of injury [20]. Type I IFNs and RANTES are known to be related to the activation and infiltration of macrophages. Thus, in order to elucidate the relative increase of macrophage population in lungs of RSV-infected mice by exposure to ISP, we examined IFN-α and β and RANTES levels in BALF prepared from RSV-infected and uninfected mice with or without the exposure (Fig. 5). On day 5 post-infection, no detectable IFN-α and β were observed in RSV-infected and -uninfected mice regardless of the exposure to ISP (Figs. 5A and B) and RANTES was less detectable in any RSV-infected and uninfected mice, either (Fig. 5C). As type I IFNs are mainly produced in early stage of microbial infection [19], we also prepared BALF on day 1 post-infection to examine the production of IFN-α and β and RANTES. On the day 1, IFN-α and β and RANTES were noticeably detected in RSV-infected mice regardless of the exposure, but they were not detectable in the RSV-uninfected mice (Fig. 5). In the RSV-infected mice, exposure to ISP reduced the levels of IFN-α and β in BALF, although the reduction of IFN-β was statistically significant but that of IFN-α was not (Figs. 5A and B). However, the exposure increased RANTES level although it was not statistically significant (Fig. 5C). ISP exposure obviously affected the levels of IFN-α and β and RANTES in RSV-infected mice, but did not produce IFN-α and β and RANTES in RSV-uninfected mice. Thus, the rise of RANTES level in the early stage of RSV infection by exposure to ISP might contribute to the rise of ratio of macrophage population in the lungs.

## 4 Discussion

Previously *S. pneumoniae* was pathologically suggested to exacerbate RSV infection not only *in vitro* but also *in vivo* [8,[10], [11], [12]]. Also, host immune response to RSV infection has been demonstrated to greatly contribute to the exacerbation of pneumonia in RSV infection [[5], [6], [7],^15^]. However, in concurrent infection by RSV and *S. pneumoniae,* the mode of exacerbation of pneumonia by *S. pneumoniae* was unclear. Recently, we found that exposure of mice to TiO_2_ nanoparticles as nonpathogenic particles exacerbates the pneumonia of RSV-infected mice, and that the exacerbation was largely responsible for the overreaction of the immune system of RSV-infected mice to TiO_2_ nanoparticles [7]. Thus, in this study, we investigated the effects of ISP as non-pathogenic particles on the severity of pneumonia in RSV-infected mice to assess a mode of exacerbation of RSV infection by concurrent infection with *S. pneumoniae.* We demonstrated that the ISP as non-pathogenic pneumococcal particles did not exacerbate pneumonia in RSV infection, but rather, it mildly reduced the severity.

In this study, *S. pneumoniae* was inactivated by formalin. However, no remaining formalin in the suspension of ISP was detected, and abnormal behavior of the mice was not observed. Histopathological analysis of lung tissues and bronchoalveolar lavage cells showed that there was no difference between RSV-uninfected mice with and without exposure to ISP (Fig. 1, Fig. 3). Thus, the significant toxicity of formalin was not observed in mice. In our RSV infection model in mice, mice were intranasally exposed to ISP at 1.0 × 10^7^ CFU/0.1 mL once daily on days 1, 3, and 5 before RSV infection. The dose of ISP used was an immunologically active amount in mice, because a sufficient TNF-α level as an initial immune response was detected in the BALF prepared from ISP-exposed mice (data not shown). Further, as shown in Table 2, the exposure to ISP noticeably increased the total number of infiltrated cells in BALF of RSV-uninfected mice. Thus, the dose of ISP was indicated to be sufficiently effective in producing an immunological action in RSV-infected mice in this study. It was confirmed that the ISP did not exacerbate pneumonia in RSV infection, even though immunological response by the ISP occurred in mice.

Significant effects were not histopathologically observed in the lungs of RSV-infected mice exposed to ISP on day 5 post-infection (Fig. 1 and Table 1). Also, the IFN-γ level in the BALF of RSV-infected mice, reflecting the exacerbation of pneumonia in RSV-infected lungs, was not significantly augmented by the exposure to ISP (Fig. 2). The total number of cells infiltrated into BALF of RSV-infected mice after exposure to ISP was not significantly higher than that in RSV-infected mice without the exposure (Table 2). These results indicated that the ISP did not exacerbate pneumonia in RSV infection. The ISP as non-pathogenic particles was found not to be a deleterious factor in pneumonia in RSV-infected mice. In concurrent infections of RSV and *S. pneumoniae,* it was suggested that live *S. pneumoniae* and its growth *in vivo* are important to exacerbate pneumonia in RSV infection. In our study, RSV infection was preceded by ISP exposure, because many healthy children and adults possess *S. pneumoniae* in the upper respiratory tract as described previously [9]. However, it may be clinically possible that *S. pneumoniae* infection occurs after RSV infection. Thus, it would be also interesting to compare the clinical and immunological effects of RSV-infected mice before and after the exposure to ISP.

As shown in Table 2, the exposure of ISP significantly reduced the ratio of lymphocytes in the total infiltrated cells of BALF while it increased the ratio of macrophages. In BALF of RSV-uninfected mice, the exposure also produced the dominant infiltration of macrophages. The increase in the macrophage population in BALF of RSV-infected mice not only without but also with the exposure to ISP might reflect the strong ability of ISP to infiltrate macrophages in mice. Some researchers have reported that macrophages are important in the host defense mechanism against live *S. pneumoniae* [19,21,22]. Even against the exposure to ISP, macrophages might play a significant role in the immunity of host defense.

In RSV infection, macrophages have been shown to be pathologically major cells that can produce IFN-γ [5]. Table 2 shows that the exposure to ISP slightly decreased the total number of infiltrated cells in the BALF of RSV-infected mice but significantly increased the ratio of macrophages in the total infiltrated cells. As a result, the substantial number of macrophages in total infiltrated cells in the BALF of RSV-infected mice with the exposure did not seem to be remarkably different from that in the RSV-infected mice without the exposure. Thus, IFN-γ levels in the BALF of RSV-infected mice might not be significantly different regardless of the exposure, as shown in Fig. 2.

The exposure to ISP reduced the virus titer in the lungs of RSV-infected mice (Fig. 3). In RSV infection, cellular immunity mediated by lymphocytes, especially T cells, plays a critical role in virus elimination, and its overreaction causes the exacerbation of pneumonia [5,6]. However, the exposure to ISP was not able to exacerbate pneumonia in RSV infection as described above. This suggested that cellular immunity was not sufficiently activated by ISP in RSV-infected mice. Also, the ISP significantly reduced the population of lymphocytes in BALF of RSV-infected mice and significantly increased that of macrophages (Table 2). Thus, it is likely that the reduction of lymphocyte populations by the exposure promoted the insufficient activation of cellular immunity as compared with the case without the exposure. Live *S. pneumoniae* has been demonstrated to promote the production of type I IFNs from macrophages [19]. However, in our study, exposure to ISP was not effective in producing IFN-α and β in BALF of RSV-uninfected mice and the levels of IFN-α and β in the BALF of RSV-infected mice were reduced by the exposure, as shown in Fig. 5. ISP was not a stimulator of type I IFN production *in vivo*. The reduction of virus titer in the lungs of RSV-infected mice by exposure to ISP was suggested not to be due to the antiviral activity of type I IFNs. RANTES has been shown to act as a chemoattractant for NK cells, which are important in viral clearance in the early stage of infection [23]. Also, it has been reported to block apoptosis of alveolar macrophages [24] and to be involved in antimicrobial activity by inducing NO in macrophages [25]. These results indicate that RANTES may indirectly possess antiviral activity. Thus, the rise of RANTES level in the BALF of RSV-infected mice with exposure to ISP might contribute to the reduction of virus titer in the lungs.

In this study, the ISP as non-pathogenic pneumococcal particles was demonstrated not to be the deleterious factor of pneumonia in RSV-infected mice, but rather it was possible that the ISP slightly ameliorated pneumonia. It has been recently reported that a higher nasopharyngeal pneumococcal density was correlated with a less severe course of disease when pneumococcus is present in patients [26]. Thus, it may be possible that ISP is useful to alleviate or retard the development of pneumonia caused by RSV infection through the avoidance of immunological overreaction by activation of cellular immunity.

## Conflict of interest

The authors declare no conflict of interest.

## Transparency document

Transparency document

## References

[1] MacDonald N.E., Hall C.B., Suffin S.C., Alexson C., Harris P.J., Manning J.A. (1982). Respiratory syncytial viral infection in infants with congenital heart disease. N. Engl. J. Med..

[2] Holberg C.J., Wright A.L., Martinez F.D., Ray C.G., Taussig L.M., Lebowitz M.D. (1991). Risk factors for respiratory syncytial virus-associated lower respiratory illnesses in the first year of life. Am. J. Epidemiol..

[3] Falsey A.R., Hennessey P.A., Formica M.A., Cox C., Walsh E.E. (2005). Respiratory syncytial virus infection in elderly and high-risk adults. N. Engl. J. Med..

[4] Graham B.S. (2011). Biological challenges and technological opportunities for respiratory syncytial virus vaccine development. Immunol. Rev..

[5] Farrag M.A., Almajhdi F.N. (2016). Human respiratory syncytial virus: role of innate immunity in clearance and disease progression. Viral Immunol..

[6] Watanabe W., Shimizu T., Sawamura R., Hino A., Konno K., Hirose A., Kurokawa M. (2010). Effects of tetrabromobisphenol A, a brominated flame retardant, on the immune response to respiratory syncytial virus infection in mice. Int. Immunopharmacol..

[7] Hashiguchi S., Yoshida H., Akashi T., Komemoto K., Ueda T., Ikarashi Y., Miyauchi A., Konno K., Yamanaka S., Hirose A., Kurokawa M., Watanabe W. (2015). Titanium dioxide nanoparticles exacerbate pneumonia in respiratory syncytial virus (RSV)-infected mice. Environ. Toxicol. Pharmacol..

[8] Nguyen D.T., Louwen R., Elberse K., van Amerongen G., Yüksel S., Luijendijk A., Osterhaus A.D., Duprex W.P., de Swart R.L. (2015). *Streptococcus pneumoniae* enhances human respiratory syncytial virus infection *in vitro* and in vivo. PLoS One.

[9] Bogaert D., de Groot R., Hermans P.W. (2004). *Streptococcus pneumoniae* colonisation: the key to pneumococcal disease. Lancet Infect. Dis..

[10] Duttweiler L., Nadal D., Frey B. (2004). Pulmonary and systemic bacterial co-infections in severe RSV bronchiolitis. Arch. Dis. Child..

[11] Bosch A.A., Biesbroek G., Trzcinski K., Sanders E.A., Bogaert D. (2013). Viral and bacterial interactions in the upper respiratory tract. PLoS Pathog..

[12] Hament J.M., Aerts P.C., Fleer A., van Dijk H., Harmsen T., Kimpen J.L., Wolfs T.F. (2005). Direct binding of respiratory syncytial virus to pneumococci: a phenomenon that enhances both pneumococcal adherence to human epithelial cells and pneumococcal invasiveness in a murine model. Pediatr. Res..

[13] Avadhanula V., Wang Y., Portner A., Adderson E. (2007). Nontypeable *Haemophilus influenzae* and *Streptococcus pneumoniae* bind respiratory syncytial virus glycoprotein. J. Med. Microbiol..

[14] Kerger B.D., Fedoruk M.J. (2015). Pathology, toxicology, and latency of irritant gases known to cause bronchiolitis obliterans disease: Does diacetyl fit the pattern?. Toxicol. Rep..

[15] Watanabe W., Shimizu T., Hino A., Kurokawa M. (2008). Effects of decabrominated diphenyl ether (DBDE) on developmental immunotoxicity in offspring mice. Environ. Toxicol. Pharmacol..

[16] de Beer F., Lagrand W., Glas G.J., Beurskens C.J., van Mierlo G., Wouters D., Zeerleder S., Roelofs J.J., Juffermans N.P., Horn J., Schultz M.J. (2016). Nebulized C1-esterase inhibitor does not reduce pulmonary complement activation in rats with severe *Streptococcus pneumoniae* pneumonia. Cell Biochem. Biophys..

[17] McKimm-Breschkin J.L. (2004). A simplified plaque assay for respiratory syncytial virus--direct visualization of plaques without immunostaining. J. Virol. Methods.

[18] Takeshita T., Watanabe W., Toyama S., Hayashi Y., Honda S., Sakamoto S., Matsuoka S., Yoshida H., Takeda S., Hidaka M., Tsutsumi S., Yasukawa K., Park Y.K., Kurokawa M. (2013). Effect of Brazilian propolis on exacerbation of respiratory syncytial virus infection in mice exposed to tetrabromobisphenol a, a brominated flame retardant. Evid. Complement. Alternat. Med..

[19] Koppe U., Högner K., Doehn J.M., Müller H.C., Witzenrath M., Gutbier B., Bauer S., Pribyl T., Hammerschmidt S., Lohmeyer J., Suttorp N., Herold S., Opitz B. (2012). *Streptococcus pneumoniae* stimulates a STING- and IFN regulatory factor 3-dependent type I IFN production in macrophages, which regulates RANTES production in macrophages, cocultured alveolar epithelial cells, and mouse lungs. J. Immunol..

[20] Lee C.-M., Peng H.-H., Yang P., Liou J.-T., Liao C.-C., Day Y.-J. (2017). C-C chemokine ligand-5 is critical for facilitating macrophage infiltration in the early phase of liver ischemia/reperfusion injury. Sci. Rep..

[21] Opitz B., van Laak V., Eitel J., Suttorp N. (2010). Innate immune recognition in infectious and noninfectious diseases of the lung. Am. J. Respir. Crit. Care Med..

[22] Visan L., Rouleau N., Proust E., Peyrot L., Donadieu A., Ochs M. (2018). Antibodies to PcpA and PhtD protect mice against *Streptococcus pneumoniae* by a macrophage- and complement-dependent mechanism. Hum. Vaccin. Immunother..

[23] Hussell T., Openshaw P.J. (1998). Intracellular interferon-gamma expression in natural kiler cells precedes lung CD8+ T cell recruitment during respiratory syncytial virus infection. J. Gen. Virol..

[24] Maghazachi A.A., al-Aoukaty A., Schall T.J. (1994). C-C chemokines induce the chemotaxis of NK and IL-2-activated NK cells Role for G proteins. J. Immunol..

[25] Villalta F., Zhang Y., Bibb K.E., Kappes J.C., Lima M.F. (1998). The cysteine-cysteine family of chemokines RANTES, MIP-1a, and MIP-1b induce trypanocidal activity in human macrophages vis nitric oxide. Infect. Immun..

[26] Vissers M., Ahout I.M., van den Kieboom C.H., van der Gaast-de Jongh C.E., Groh L., Cremers A.J., de Groot R., de Jonge M.I., Ferwerda G. (2016). High pneumococcal density correlates with more mucosal inflammation and reduced respiratory syncytial virus disease severity in infants. BMC Infect. Dis..

